# Influence of Oxidation Degree on the Physicochemical Properties of Oxidized Inulin

**DOI:** 10.3390/polym12051025

**Published:** 2020-05-01

**Authors:** Franklin Afinjuomo, Paris Fouladian, Thomas G. Barclay, Yunmei Song, Nikolai Petrovsky, Sanjay Garg

**Affiliations:** 1 Pharmaceutical Innovation and Development Group, University of South Australia, Adelaide 5000, Australia; olumide.afinjuomo@mymail.unisa.edu.au (F.A.); paris.fouladian@mymail.unisa.edu.au (P.F.); tom.barclay@unisa.edu.au (T.G.B.); May.Song@unisa.edu.au (Y.S.); 2 Vaxine Pty. Ltd., Adelaide 5042, Australia; nikolai.petrovsky@flinders.edu.au; 3 Department of Endocrinology, Flinders University, Adelaide 5042, Australia

**Keywords:** oxidized inulin, periodate oxidation, aldehyde content, degree of oxidation and physicochemical properties

## Abstract

This paper reports the oxidation of inulin using varying ratios of sodium periodate and the characterization of the inulin polyaldehyde. The physicochemical properties of the inulin polyaldehyde (oxidized inulin) were characterized using different techniques including 1D NMR spectroscopy, ^13^C Nuclear magnetic resonance (NMR), Fourier transform infrared spectroscopy (FTIR), thermal gravimetric analysis (TGA), differential scanning calorimetric (DSC), ultraviolet-visible spectroscopy (UV), and scanning electron microscopy (SEM). The aldehyde peak was not very visible in the FTIR, because the aldehyde functional group exists in a masked form (hemiacetal). The thermal stability of the oxidized inulin decreased with the increasing oxidation degree. The smooth spherical shape of raw inulin was destructed due to the oxidation, as confirmed by the SEM result. The ^1^HNMR results show some new peaks from 4.8 to 5.0 as well as around 5.63 ppm. However, no aldehyde peak was found around 9.7 ppm. This can be attributed to the hemiacetal. The reaction of oxidized inulin with tert-butyl carbazate produced a carbazone conjugate. There was clear evidence of decreased peak intensity for the proton belonging to the hemiacetal group. This clearly shows that not all of the hemiacetal group can be reverted by carbazate. In conclusion, this work provides vital information as regards changes in the physicochemical properties of the oxidized inulin, which has direct implications when considering the further utilization of this biomaterial.

## 1. Introduction

Inulin is a natural linear polysaccharide obtained from plants, edible fruits, and vegetables, as well as cereals such as chicory root, Jerusalem artichoke, banana, leeks, and garlic [1,2]. Aside from plant extraction, inulin can also be obtained from genetically modified potatoes and by enzymatic production [3,4]. Inulin is made up of about 2–60 linear chains of β-(2,1) fructose units with a glucose unit attached at the reducing end [5,6]. The nutritional benefit of inulin as dietary fibers and probiotics makes this polysaccharide an important part of human diets, particularly in America and Europe [1]. In the food industries, inulin has been extensively used as a sugar and fat replacement ingredient [7,8,9,10,11,12], a nutritional ingredient [13,14], textural modifier [7,15,16], and organoleptic improvement [17]. The fact that inulin glycosidic bonds are indigestible to humans makes them good candidates for dietary fiber with prebiotic properties [1,18,19,20,21,22,23,24]. Inulin is gaining attention from the biotechnology industries because it is a non-toxic, biodegradable, compatible, cheap, and versatile substance with diverse and numerous promising applications [25]. The functionalization and modification of inulin offer enormous possibilities to transform this material into derivatives that can be exploited for drug delivery biomaterials. Modified inulin has also been used for different delivery systems such as nanoparticles, liposomes, chelating complexes, hydrogels, prodrugs, micelles, and microparticles [25,26,27,28,29,30,31,32,33].

The use of periodate oxidation to obtain new modified materials has been reported for many natural polysaccharides such as alginates, cellulose, pectin, dextran, xanthan gum, and xylan [34,35,36,37,38,39]. Few reports on inulin oxidation with periodate are well documented in the literature [40]. The use of periodate to oxidize inulin result in modified materials with aldehyde groups, which can serve as a hook or anchor to attach drugs [40] as well as the formation of hydrogels [41]. Typically, the oxidation results in the cleavage of the C_3_–C_4_ bond in the building units of fructose, which results in the formation of two aldehyde units that are present in several masked forms [40,42]. The aldehydes formed during inulin oxidation react with the neighboring C_6_ hydroxyl group, which eventually results in stable hemiacetal formation [43,44]. In addition, work from Schacht et al. reported that due to the formation of stable hemiacetal between C_3_ and the hydroxyl group attached to C_6_, the inulin derivative is left with only the aldehyde group at C_4_ group for further reaction [40]. The structural elucidation of such polysaccharide dialdehydes remains a big challenge. The amount of drug that can be attached to oxidized inulin during the preparation of macromolecules [40] as well as the properties of hydrogel obtained from oxidized materials depends on the proper elucidation of the structural nature of oxidized inulin. However, there is little or no report on the characterization and influence of the oxidation on the structural, morphological, solubility, and thermal properties of the oxidized inulin. Previous work with oxidized inulin looked at using this modified material for the coupling of procainamide, enzyme-immobilizing ability, and hydrogel formation after reacting with a crosslinker [40,41,45]. To address the gap, this study highlights the influence of oxidation degree on the final modified product. To do this, four samples of the modified inulin with different degree of oxidation were synthesized. The influence of the varying ratio of oxidizing agents on the physicochemical properties of the modified samples was investigated using different techniques such as Fourier transform infrared spectroscopy (FTIR), SEM, ^1^HNMR, thermal gravimetric analysis (TGA), and XRD. In order, to provide adequate information on the complicated oxidized inulin structure and behavior, we have combined the use of NMR techniques to provide an answer by attacking the C_3_ and C_4_ masked aldehyde with a nucleophilic agent *ter*-butyl carbazate. We aimed to investigate how this oxidation will alter the physicochemical properties of inulin, which is highly important for formulation scientists when designing a drug delivery system where oxidized inulin will be used as raw or intermediate materials.

## 2. Materials and Methods

Inulin was purchased from Sigma and using the end-group analysis with ^1^HNMR, the average chain length (DPn) 39.2 and MWn = 6381.7 was obtained [46]. Other chemicals such as sodium metaperiodate, *tert*-butyl carbazate (tBC), ethylene glycol, and trinitrobenzene sulfonic acid were purchased from sigma. The dialysis bag was CelluSep T4 25 mm flat width 12,000 MWCO purchased from Fisher Biotec Australia. The deuterated water (D_2_O) and DMSO (DMSO-d6) for NMR was purchased from Cambridge Isotope Laboratories. All chemicals used for this work were analytic grade chemicals and used as received without any further modification or purification.

### 2.1. Oxidation of Inulin to Inulin Aldehyde Derivative

Inulin was oxidized with variable amounts of sodium periodate in order to obtain modified inulin with different degrees of oxidation (DO). Then, 1.2 g of inulin was dissolved in 10 mL of water and was oxidized with varying concentrations of sodium periodate (60–360 mg) to yield oxidized inulin with theoretical oxidations from 5% to 30%. The oxidation was carried out for five hours at room temperature and in the dark. The oxidation was quenched by using an excess amount of ethylene glycol, and the resulting solution after the oxidation was purified by dialysis against MilliQ water for 3 days. The final solution was subsequently lyophilized using a freeze dryer. The obtained white fluffy product was referred to as Oxi5, Oxi10, Oxi20, and Oxi30 based on theoretical oxidation rates of 5%, 10%, 20%, and 30% respectively. The yield obtained range was from 70.2% to 86.3%

### 2.2. Proton Nuclear Magnetic Resonance (^1^H NMR) Spectroscopy)

The proton NMR spectra for the oxidized inulin and the product obtained after reacting with carbazate was acquired using the Bruker Avance III 500 NMR, Bruker NMR. Briefly, about 30 mg of modified inulin was dissolved in 0.5 mL of D_2_O after heating for about 2 min at 80 °C; then, a total of 64 scans was obtained for each sample using the 5 mm NMR probe. In addition, carbon NMR spectra of raw inulin and oxidized inulin in D_2_O were obtained with Bruker spectrometer (Bruker, Wissembourg, France) using standard pulse sequences. Then, all the spectra obtained were analyzed using the ACD NMR software.

### 2.3. Determination of Aldehyde Content Using TNBS Assay (Carbazate-TNBS Assay)

Furthermore, the percentage degree of oxidation for all the oxidized inulin samples was measured using the trinitrobenzene sulfonic acid (TNBS) assay method. The formation of carbazones by oxidized sugar after reacting with carbazate is similar to the mechanism of hydrazides forming hydrazones after reacting with polyaldehyde sugar. Therefore, this reaction allows the quantification of the aldehyde content in oxidized inulin [47,48]. Briefly, about (0.5 mL, 0.6% oxidized inulin sample) dissolved in 1% aqueous trichloroacetic acid solution was allowed to react for 24 h at room temperature with excess tBC (0.5 mL, 30.0 mM), which was also dissolved in 1% aqueous trichloroacetic acid solution. Then, a volume of about 200 μL from the mixture was transferred into a centrifuge tube containing 1 mL of aqueous trinitrobenzene sulfonic acid (TNBS) (85.50 mM dissolved in phosphate buffer with pH 8). Then, this mixture was reacted for another 1 h at room temperature resulting in the formation of a red color mixture. The resulting solution was further diluted with aqueous hydrochloric acid (0.5 N) and finally, the unreacted portion of tBC was followed by measuring the absorbance of the stable yellow colored complex derivative formed at 334 nm using UV/VIS spectrophotometer. The standard curve of aqueous tBC was prepared (3–30 mM), which allows the determination of unreacted tBC in the experimental samples. Water and 1% aqueous trichloroacetic acid solution in a ratio of 1:1 was used as a blank in the UV experiment. Three replicates were performed for all the titration experiments. The degree of oxidation of inulin was calculated from the amount of tBC consumed in the reaction.

### 2.4. Fourier Transform Infrared Spectroscopy (FTIR)

About 2 mg each of dried raw inulin and the modified samples Oxi5, Oxi10, Oxi20, and Oxi30 samples with different degrees of oxidation was properly mixed with 100 mg of FTIR grade KBr powder to form a pellet; then, the FTIR spectra were obtained using a Shimadzu IR Prestige-21 FTIR 8400 spectrophotometer (Kyoto, Japan). All the spectra were obtained after 128 scans in the range of 4000–400 cm^−1^.

### 2.5. TGA

The freeze-dried samples Oxi5, Oxi10, Oxi20, and Oxi30 as well as raw inulin were subjected to thermal analysis using a TA Instrument (Thermogravimetric Analyzer Discovery TGA 550, New Castle, DE, USA). About 6 ± 0.1 mg of the oxidized inulin and raw inulin samples was subjected to heating under a nitrogen atmosphere at a heating rate of 10 °C/min from room temperature to about 500 °C. Finally, the weight loss of the raw inulin and oxidized samples and the derivative thermogravimetry (DTG) were recorded as a function of time and temperature. 

### 2.6. UV Spectrophotometry and Solubility

About 20 mg of raw inulin was suspended in MilliQ water, and this was heated to about 60 °C for about 5 min to ensure complete dissolution. The sample was allowed to cool to room temperature before adding 6 mg of NaIO_4_. Then, about 1 mL of the mixture was transferred into a 1 mL UV cuvette, and the concentration of periodate consumed during the oxidation reaction was monitored using UV spectrophotometry by measuring the absorbance of the periodate left in the cuvette at 290 nm. For the solubility experiment, about 200 mg of each sample was added into 6 mL of phosphate-buffered saline (PBS; pH 7.4), and the mixture was shaken vigorously.

### 2.7. Scanning Electron Microscopy (SEM)

The morphology of raw inulin and the oxidized sample was investigated using a Zeiss Merlin Field Emission Gun Scanning Electron Microscope (Germany). The freeze-dried samples of the raw and modified inulin were mounted on a double-sided tape before sputter coating with platinum (approximately 5 nm). Then, the micrographs were captured using an accelerating voltage of 2–5 kV.

### 2.8. X-ray Diffraction (XRD)

The change in the crystallinity of raw inulin after modification was investigated by measuring the X-ray diffraction patterns of both the raw inulin and the modified samples using Empyrean, Malvern panalytical XRD equipment (Empyrean XRD Malvern, Worcestershire, UK), which was coupled with a graphite crystal monochromator with a filter radiation of Cu-K_α1_ (λ = 1.5406 Å) at 30 kV and 30 mA. The diffraction angle for the analysis (2θ) was from 5° to 50° with a scanning speed set at 1.2°/min.

### 2.9. Differential Scanning Calorimetric (DSC) Analysis

The thermal analysis of the raw inulin and all the oxidized samples was examined using the Discovery DSC TA Instrument (model Discovery DSC 2920, New Castle, DE, USA). Raw inulin and all the oxidized inulin samples were accurately weighed (2 mg of each sample) and placed in a sealed aluminum hermetic pan. The samples were subsequently heated from room temperature to 250 °C in a nitrogen atmosphere at a rate of 10 °C/min.

## 3. Results

### 3.1. Synthesis and Degree of Oxidation

**Scheme 1:**
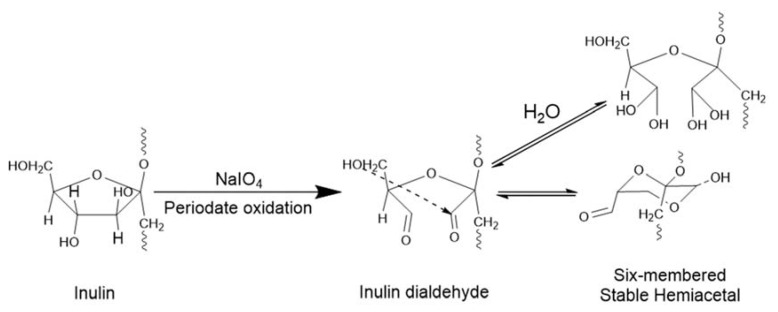
Showing oxidation of inulin.

As reported in the method section, inulin was modified using varying ratios of sodium periodate, which ultimately resulted in the formation of aldehyde inulin with different degrees of oxidation. Generally, fructan such as inulin with a vicinal diol moiety can be converted to highly reactive intermediate with aldehyde functional group by oxidation with sodium periodate (Scheme 1). The oxidation introduces aldehyde groups into the inulin structure, which then readily reacts with nucleophilic agents such as hydrazide to form hydrazones and amines to form a Schiff base. This oxidation reaction, as previously mentioned, results in the cleavage of the C_3_–C_4_ bond of the furanose ring and the creation of aldehyde groups attached to these carbon atoms (Scheme 1). Available evidence suggests that these aldehyde functional groups can react with the hydroxyl group within and around the oxidized inulin to form either intra or inter hemiacetal groups [43,49]. Inulin aldehyde can exist in solution as either hydrated and/or hemiacetal. The hemiacetal and hydrated forms depicted in Scheme 1 are reversible and exist in equilibrium with the free aldehyde form. The modified inulin with an aldehyde functional group finds usefulness in hydrogel crosslinking and the successful attachment of drugs via conjugations. The time course of inulin oxidation with periodate was followed using UV spectrophotometry by measuring the absorbance of sodium periodate consumed as the reaction proceeds at 290 nm. The result indicates that the oxidation reaction proceeds rapidly during the first three hours and subsequently slows down during the remaining two hours (Figure S1).

As reported in the method section, the degree of oxidation was quantified by using *t*BC and TNBS assay, and the result shows that with the increase in the ratio of sodium periodate used during the oxidation reaction, there was an increase in the degree of oxidation from 2.8% to 19.4% (Table 1). This was far below the theoretical degree of oxidation.

The difference between the experimental result and theoretical result is clearly because not all of the aldehyde functional group participated in the reaction with the carbazate [38]. This was further supported by our NMR result.

### 3.2. FTIR

**Figure 1:**
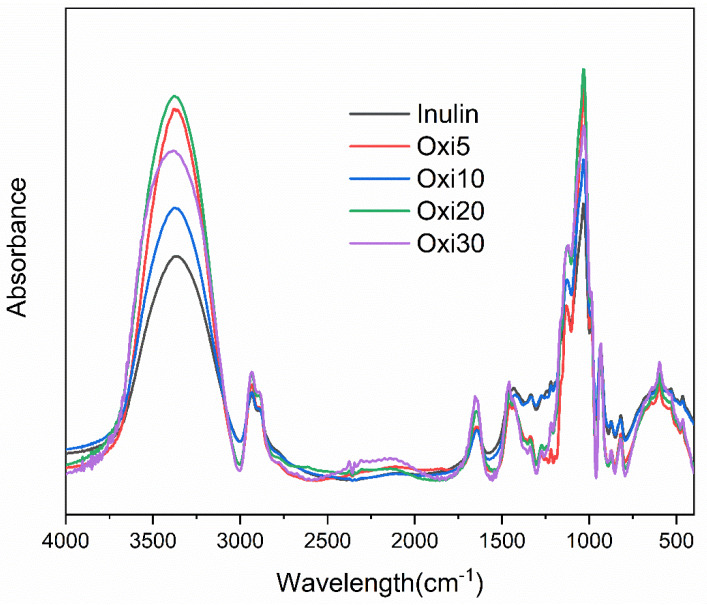
Fourier transform infrared spectroscopy (FTIR) spectra for all the modified inulin samples and raw inulin.

**Figure 2:**
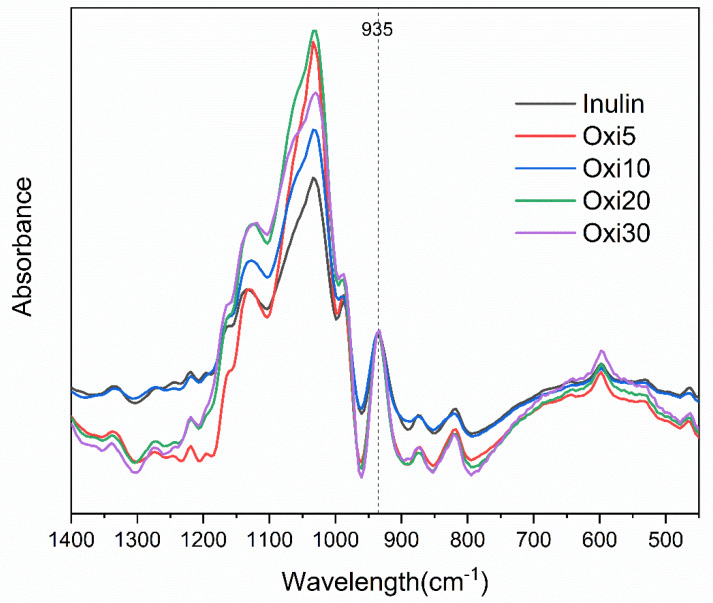
FTIR spectra for all the modified inulin samples and raw inulin from 1400 to 450 cm^−1^.

The unmodified raw inulin shows a strong OH stretching band around 3363 cm^−1^, aliphatic CH_2_ stretching at 2926 cm^−1^, and a band at 1028 cm^−1^ which is ascribed to COC bending [50]. Inulin additional bands include those seen at 1170 cm^−1^, 934 cm^−1^, 873 cm^−1^, and 817 cm^−1^, and two shoulders at 1132 cm^−1^ and 986 cm^−1^. The band at 935 cm^−1^ can be attributed to the inulin β2→1 glycosidic bond [51], and as the DO increases, the peak area of this band was decreasing, which possibly suggests chain degradation. However, it is difficult to distinguish any difference between the spectra of raw inulin and oxidized inulin, with both looking similar (Figure 1). As reported for dextran with aldehyde groups, the band corresponding to the aldehyde group after oxidation is expected around 1730 cm^−1^ [38]. However, a similar peak was not very visible in the FTIR spectra of all the oxidized inulin samples in this work. The explanation for this is that the aldehyde exists in a masked form (hemiacetal) [38,52] as well as hydrated aldehyde [52] and presumably, the FTIR is not powerful enough to detect the masked aldehyde functional group in this case. In addition, the low and mild level of oxidation may likely result in a slight modification, which may be difficult to detect with FTIR (Figure 2). The inability of FTIR to detect masked aldehyde in other oxidized carbohydrates such as sugar has been previously reported [34].

### 3.3. Scanning Electron Microscope (SEM) Analysis

**Figure 3:**
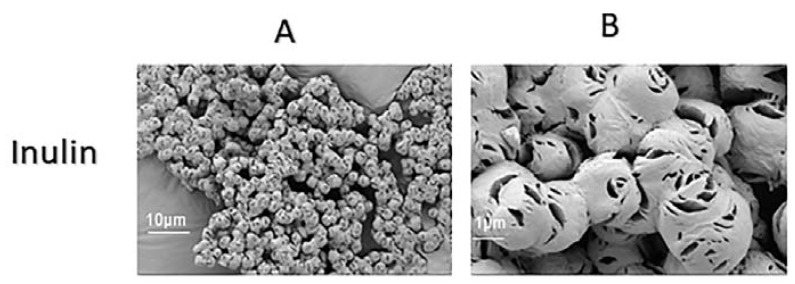
SEM images of raw inulin at two different magnification sizes: 10 μm (**A**) and 1 μm (**B**).

**Figure 4:**
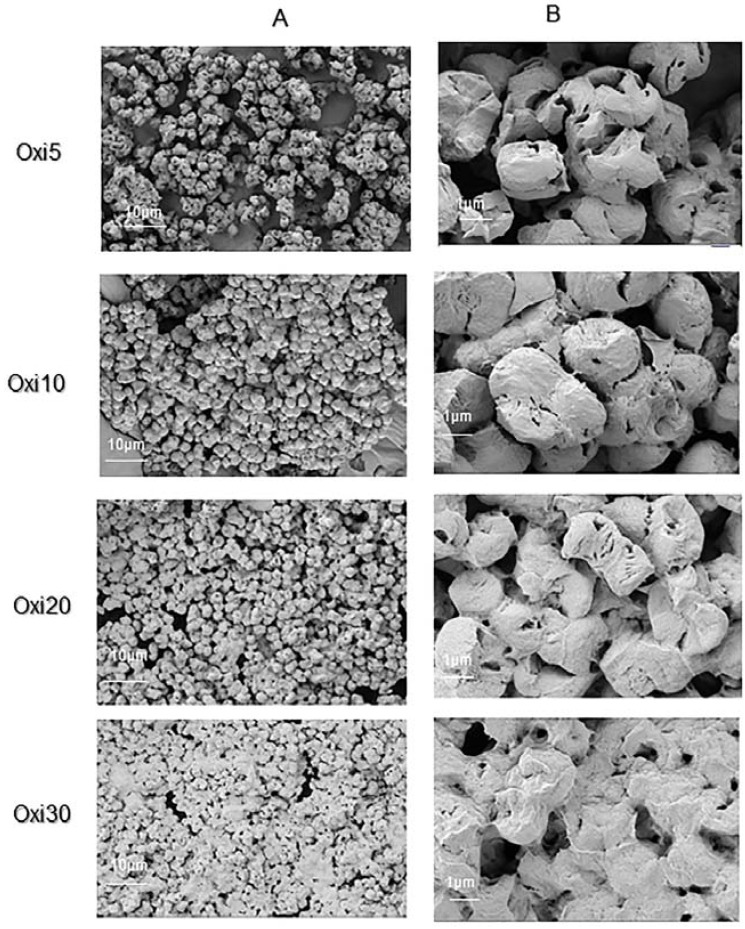
SEM of oxidized inulin samples at two different magnification sizes: 10 μm (**A**) and 1 μm (**B**).

Figure 3A,B shows the SEM of the native inulin; as reported in the literature, the native unmodified inulin appears as a cluster of spherical smooth aggregates, which is expected for spray-dried powders [53,54]. In contrast to inulin, there was a remarkable change in the surface structure of all the modified inulin samples, irrespective of the degree of oxidation (Figure 4A,B). The gradual change in surface morphology might be attributed to the fact that the oxidizing agent diffuses from the surface into the inulin microcrystal. From the SEM, as the oxidation progress, there is clear evidence of surface defects such as the conglutination of the inulin aggregates. In addition, the oxidized samples are characterized by the gradual destruction of the smooth spherical shape of inulin, possibly suggesting that oxidation starts at the surface [55]. As the ratio of sodium periodate used for the oxidation increases, there was evidence of the smooth surface of inulin changing to the rough surface characterized by larger pores compared to native inulin.

### 3.4. TGA/DTG

**Figure 5:**
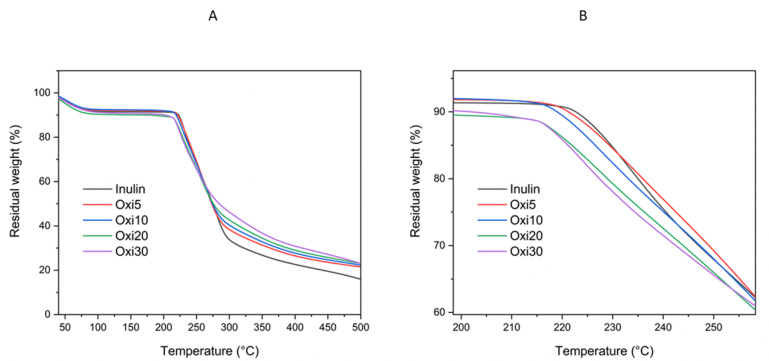
(**A**,**B**) TGA thermograms of unmodified raw inulin and Oxi5, Oxi10, Oxi20, and Oxi30 samples.

**Figure 6:**
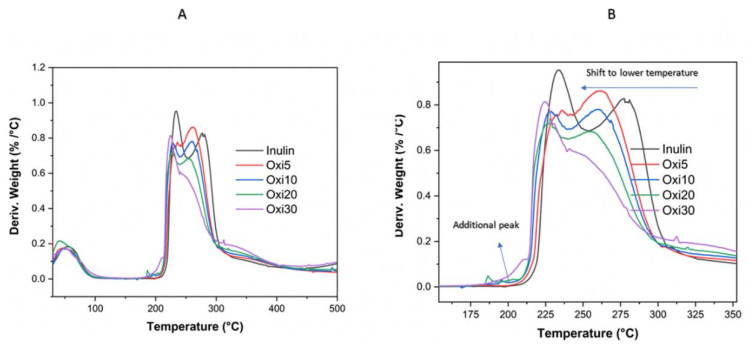
(**A**,**B**) Thermograms of unmodified raw inulin and Oxi5, Oxi10, Oxi20, and Oxi30 samples.

TGA was used to investigate the impact of increasing the degree of oxidation by using varying amounts of sodium periodate on the thermal properties of the modified inulin derivatives. The DTG graph was also plotted to help with the interpretation of the TGA analysis. The raw inulin, as shown in Figure 5, demonstrated a familiar three-stage weight loss similar to reports from the literature [56,57]. The first weight loss before 100 °C can be attributed to the loss of water and moisture followed by two-stage weight loss from 219 to 323 °C, as shown in Figure 5 and Figure 6. The second and third weight loss of inulin from the TGA investigation can be attributed to the degradation of the backbone and inulin combustion, respectively [56]. This weight loss pattern was similar for all the oxidized samples (oxi5, Oxi10, Oxi20, and Oxi30). All the oxidized inulin samples exhibited slight and similar weight loss (8.0%–9%) between room temperature and 100 °C due to the loss of adsorbed water in the samples. The onset of degradation temperature for all the oxidized inulin samples was lower than the raw inulin sample. The onset of degradation was 219, 215, 214, 212, and 210 °C for raw inulin, Oxi5, Oxi10, Oxi20, and Oxi30, respectively. The likely reason for this can be attributed to the breaking of sugar rings during the oxidation reaction, which are then reconnected through relatively weak hemiacetal bonds and degradation of the inulin structure, which is reflected in a decrease in molecular weight [34,38]. As the content of aldehyde group in the modified inulin increases, there is a shift of degradation temperature toward lower values. The thermal stability of the oxidized inulin decreased with the increasing DO of the samples, which can be attributed to depolymerization of the inulin chain during the oxidation reaction [58]. This observation and similar outcomes have been reported for oxidized sugars such as dextran, starch, cornstarch, and pea starch [38,59,60]. The slight and limited change in the decomposition temperature can be attributed to the fact that during oxidation, the inulin backbone might remain intact, as the oxidation affects the rings that hang off the backbone. Unlike other polysaccharides where the rings are part of the backbone, for inulin, the linear structure resembles the polyethylene oxide backbone [24]. Furthermore, there is a distinctive difference in the residual mass after the TGA experiment. The oxidized samples residual weight was slightly higher compared to inulin. The oxidized samples Oxi5, Oxi10, Oxi20, and Oxi30 at the end of the thermal analysis had 23.13%, 23.1%, 22.34%, and 21.58% of residual weight compared to 16.03% for inulin. This can be attributed to the hemiacetal chemistry on the surface of the oxidized samples, which possibly forms a char layer of protection and will prevent further weight loss during the TGA [61].

The significant changes in the thermal behavior of the raw inulin when compared to the oxidized inulin are more clearly seen in the DTG curves. The decomposition temperature of oxidized inulin shifted to a lower temperature with an increase in the aldehyde content. Above 170 °C, there is a slight change in the DTG of the oxidized samples in Figure 6A,B within the range of range 170–210 °C, which is not evident in the raw inulin. The peak is just beginning to show in OXI5 and becomes enlarged as the degree of oxidation increases. Thereafter, this is followed by the main decomposition process, whose pattern is also clearly influenced by the oxidation degree. It is important to explain that the reduction in the onset of degradation temperature with an increase in oxidation degree follows a well-defined pattern. In conclusion, oxidation causes a reduction in the degradation temperature.

### 3.5. DSC

**Figure 7:**
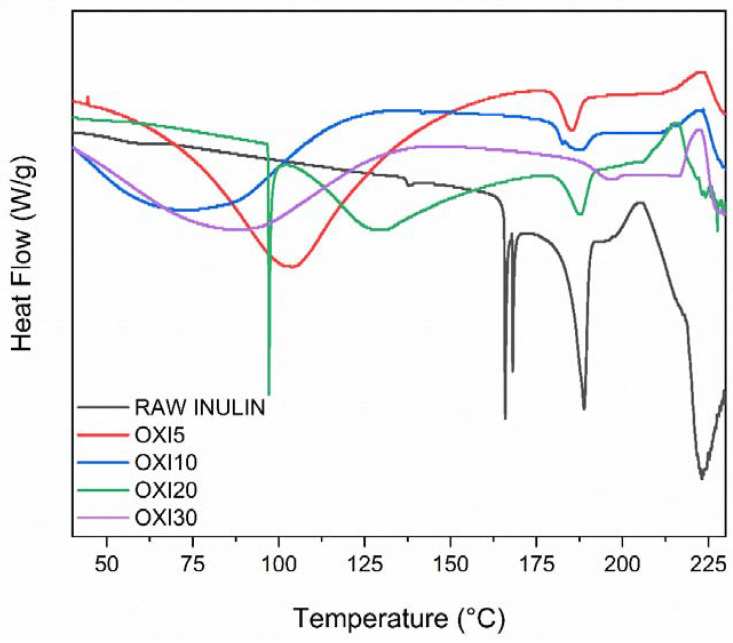
Differential scanning calorimetric (DSC) of raw inulin and all the modified inulin samples.

From Figure 7, the DSC thermogram of raw inulin shows endothermic peaks between 165 and 188 °C, which can be attributed to the melting point of different size isoforms within the polymer sample [57,62]. A previous literature report by Ronkart et al. reports that two endothermic peaks were observed during inulin DSC investigations due to the melting point of different inulin crystals within the sample [63]. Furthermore, Blecker et al. reported the melting point of inulin in the range of 165 to 195 and a shift toward higher temperature for inulin biopolymer with an increasing average polymerization degree (*DP*) [64]. The DSC of raw inulin closely matches those from previous reports in the literature [64]. After the oxidation of raw inulin with sodium periodate, there is evidence of two broad endothermic peaks in Oxi5, Oxi10 Oxi20, and Oxi30. This first broad peak between 50 and 125 °C can be attributed to the evaporation of water from the modified samples. The second peak between 184 and 189 °C may be attributed to the inulin melting point. The oxidation as expected will degrade inulin with clear evidence of the two peaks at 165, 168 °C disappearing in the modified samples. The absence of two endothermic peaks that are seen in the raw inulin between 166 and 168 °C possibly supports the assumption that oxidation degrades some fraction of the isoform unit, which is indicative of a change in the original semicrystalline structure. The changes and altered behavior in the DSC thermogram support the fact that inulin oxidation by periodate destroyed the order structure of inulin. The change in the endothermic transition due to the change in the crystallinity is consistent with the result obtained from the XRD.

### 3.6. XRD

**Figure 8:**
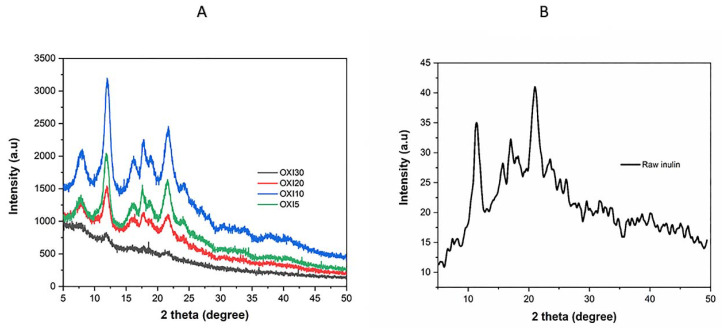
(**A**) XRD spectra of Oxi5, Oxi10, Oxi20, and Oxi30 samples and (**B**) raw inulin.

The X-ray diffraction experiment allows the measurement of the crystallinity of the raw inulin and oxidized samples (Figure 8A,B). Inulin with higher DP has been reported as semicrystalline materials [65]. The X-ray diffraction of native inulin shows strong peaks at 12°, 16.4°, 17.7°, 21.4°, 23.8°, and 37.37°, which are characteristic of semicrystalline inulin [66]. However, the mild oxidation results in a significant increase in the degree of crystallinity for both Oxi5 and Oxi10. With a further increase in oxidation, the semicrystalline nature of raw inulin was converted to amorphous material as shown in Oxi20 and Oxi 30. The oxidation of inulin results in the disruption of the inulin order structure, which promotes the rearrangement of the structure from semicrystalline to amorphous. The result of the XRD show two opposite results. For Oxi5 and Oxi10, the intensities of the peaks obtained for modified inulin are higher than those of native inulin, resulting in sharper peaks. Mild oxidation (Oxi5 and Oxi10) causes rearrangement of the inulin chain, which possibly gives modified samples with more crystalline structure, as demonstrated from the X-ray patterns. However, as the periodate concentration was enhanced further, the modified inulin obtained changed from semicrystalline structure to amorphous. As the sodium periodate concentration increases, the outcome was opposite to the mild oxidation. The higher concentrations of sodium periodate reduced crystallinity, which was due to a likely degradation of the crystalline region, and this ultimately results in the formation of amorphous substances [67]. For the Oxi20 and Oxi30, the sugar ring is likely destroyed or changed by the oxidation reaction. Similar results were obtained for oxidized starch [68] and cellulose [69]. This change may be a consequence of the oxidation process destroying the crystalline regions on the surface of the inulin with an increasing ratio of sodium periodate.

### 3.7. ^1^H and ^13^C NMR Analysis

**Figure 9:**
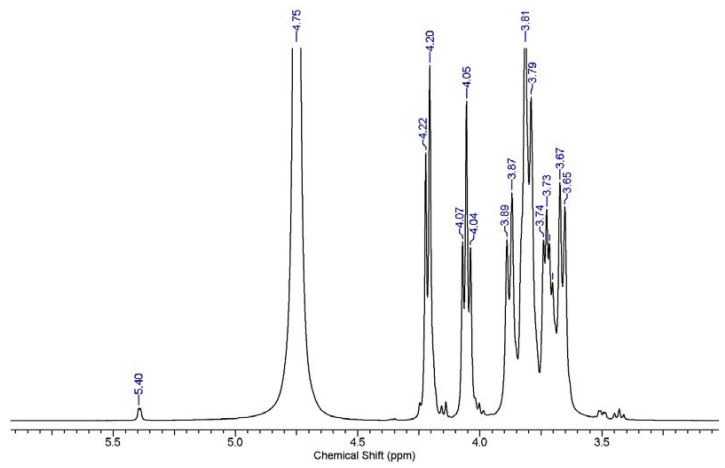
^1^HNMR of raw inulin.

**Figure 10:**
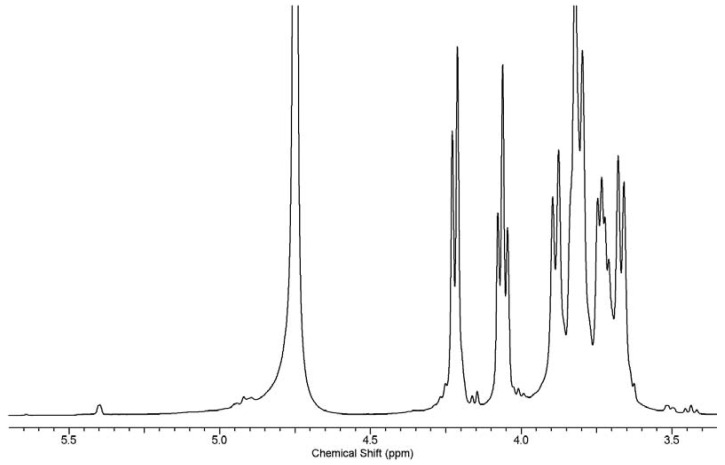
^1^HNMR of oxidized inulin (oxi5).

**Figure 11:**
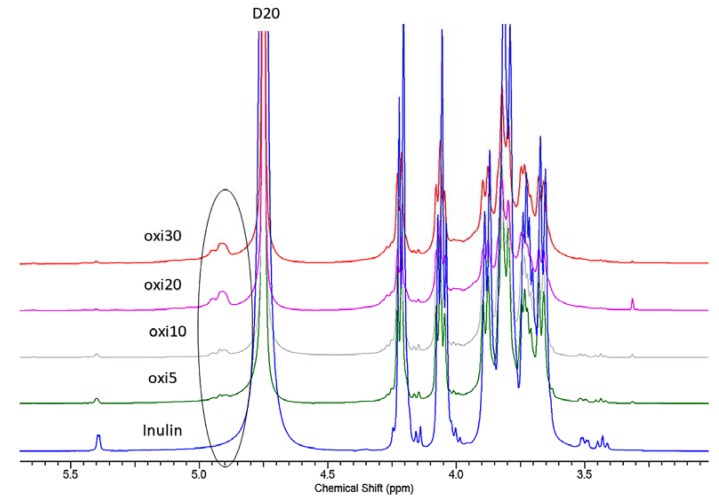
^1^HNMR of all oxidized inulin and the raw inulin.

In the case of inulin oxidation, as reported previously, the C3 aldehyde forms a particularly stable six-membered hemiacetal structure, while C4 remains as an aldehyde functional group. Essentially, this means that only C_4_-attached aldehyde is available to react, and the hemiacetal structures will prevent the C_3_ aldehyde from reaction with nucleophilic agents. The proton ^1^HNMR of raw inulin shows an anomeric glucose signal at 5.40 ppm with low intensity and the fructose signal between 3.60 and 4.25 ppm (Figure 9). The results obtained are similar and consistent with reports in the literature [46,70]. In contrast, the masked aldehyde derivative in this work was confirmed by the presence of new peaks with low intensity between 4.92–5 ppm and 5.64 ppm in the ^1^HNMR spectra of the oxidized inulin (Oxi5–Oxi30) (Figure 10 and Figure 11) Supplementary Figures S2–S8. The absence of an aldehyde functional peak around 9.7 ppm can be attributed to the formation of hemiacetal by the aldehyde group and a neighboring hydroxyl group from inulin [38,40].

The new peaks are attributed to the formation of the hemiacetal. By varying the ratio of the oxidizing agents (increased), it was found that the hemiacetal peak intensity also increased (Figure 11).

**Figure 12:**
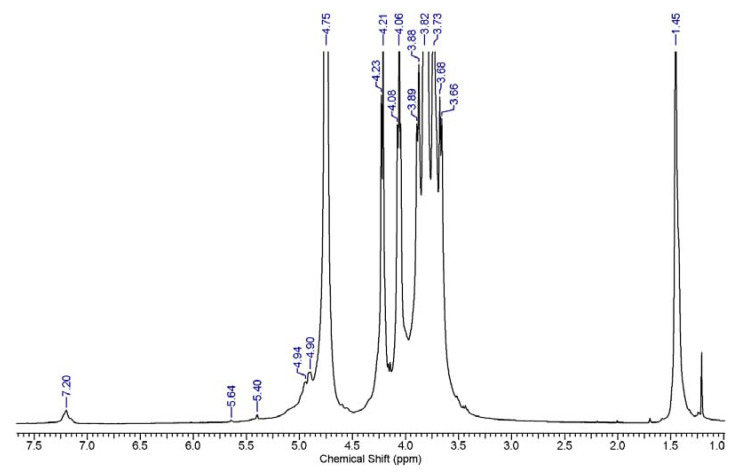
^1^HNMR of inulin conjugate with *tert*-butyl carbazate (tBC).

**Scheme 2:**
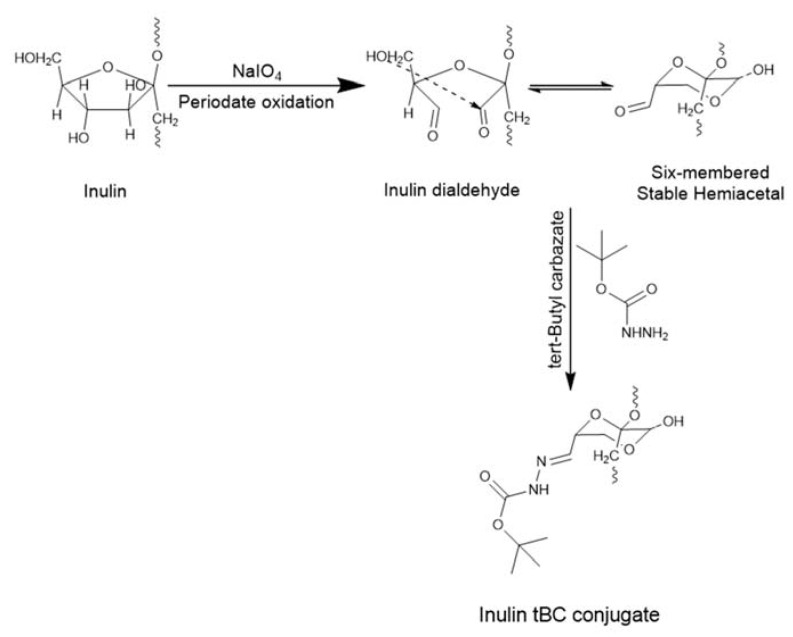
Showing the reaction between oxidized inulin and tBC.

**Figure 13:**
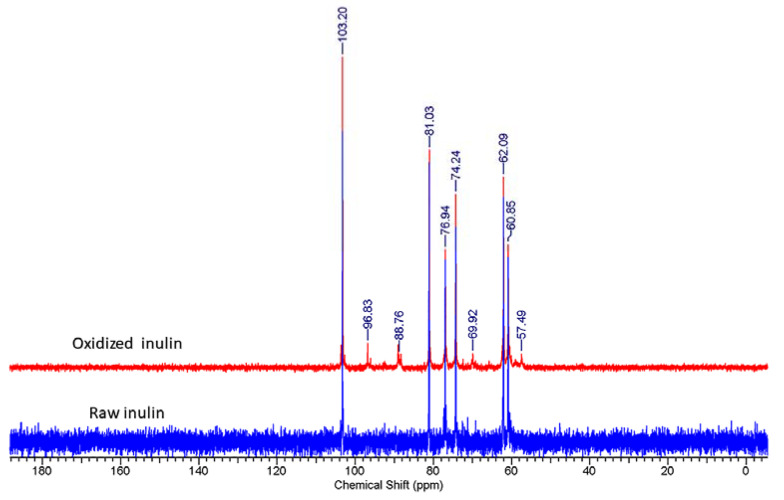
^13^C NMR spectra of inulin and oxidized inulin.

The reaction of the masked aldehyde on C_3_ and C_4_ of the oxidized inulin with excess carbazate shows that not all hemiacetal peaks can be reverted by carbazate. There was evidence of a decrease in the peak intensity for the proton corresponding to the hemiacetal group the final inulin–tBC conjugate (Figure 12). The ^1^HNMR results suggest that not all the peaks from the hemiacetal were reverted after reacting with carbazate. The reaction of tBC (cabazate with the oxidized inulin) produced a carbazone conjugate (Scheme 2). In the ^13^C NMR spectra of inulin, all of the chemical shifts are comparable to reported data from the literature with prominent shifts for C_1_–C_6_ carbons listed as follows (C1 60.83 ppm, C2 103.19 ppm, C_3_ 76.91 ppm, C_4_ approximately 74.20 ppm, C_5_ approximately 81.01 ppm, and C6 approximately 62.06 ppm) of fructosyl residue due to fructose repeated units (Figure 13). In addition, the ^13^C NMR spectra of oxidized inulin show the low intensity of a new peak along with the peaks from the raw inulin, which further confirms changes in the chemical structure. The likely reason for this low intensity is that the oxidized inulin still contains a mixture of the raw inulin as well as oxidized inulin. This result from this work, as well as the report from Schacht et al. [49] which observed the preferential reaction of the aldehyde group at position C_4_ only, will require further investigation in the future.

### 3.8. Solubility

**Figure 14:**
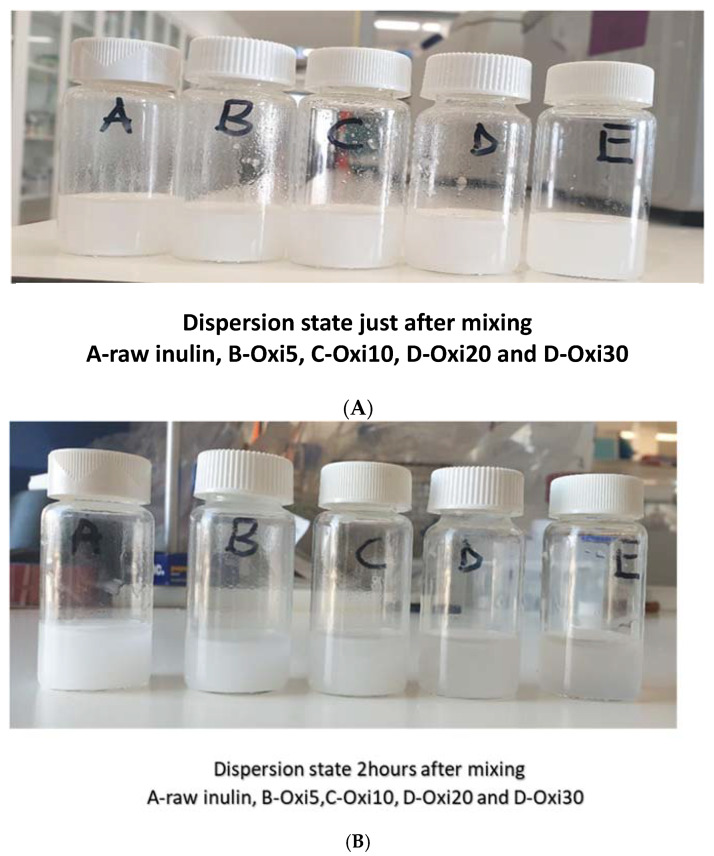
(**A**) Dispersion of inulin and the modified sample immediately after mixing in PBS; (**B**) Dispersion of inulin and the modified sample after mixing for two hours in phosphate-buffered saline (PBS).

Figure 14A,B clearly show the dispersion photograph of the raw inulin and all the oxidized samples. From the photo, there was a slight change in the appearance of the modified inulin with an increase in the degree of oxidation. All of the samples just after mixing with the PBS solution show the formation of cloudy dispersion (Figure 14A). However, after mixing for 2 h, especially for the Oxi20 and Oxi30, there was a change from cloudy solution to a transparent solution. The modified samples show better solubility compared to the raw inulin. The likely reason for the change in solubility can be attributed to a decrease in the molecular weight [38] and the slight change in the crystallinity from semicrystalline to amorphous materials. The introduction of the aldehyde functional group into the raw inulin can also facilitate improve solubility compared with the raw inulin. From Figure 14A,B, as the degree of oxidation increases, the solubility of the new biomaterials in PBS also improves. This finding clearly shows that the oxidation of raw inulin can impart improved solubility on the modified samples. Improved solubility at low concentrations with oxidized polysaccharides such as starch and cellulose has been reported in the literature [71,72]. This result corroborates the previous investigation. The reason for the aqueous solubility was attributed to enhanced hydrophilicity due to the new hydrophilic carbonyl functional group as well as disruption of the hydrogen bond, which further facilitates the entry of water into the amorphous areas [71].

## 4. Conclusions

Inulin dialdehyde derivative with varying content of an aldehyde functional group was synthesized via oxidation reaction with sodium periodate. The periodate oxidation allows the introduction of a highly reactive aldehyde functional group into inulin C_3_–C_4_. From the TNBS assay result between a 2.82.8 ± 0.36% and 19.4 ± 1.38% degree of oxidation was obtained after modifying with sodium periodate. Furthermore, from the results obtained, the modification has effects on the physicochemical properties such as morphology, thermal stability, and solubility. This work will help with the use of oxidized inulin as biomaterials for hydrogel crosslinking and the attachment of drugs.

## Figures and Tables

**Table 1:** Degree of oxidation for the oxidized inulin. DO: degrees of oxidation, TNBS: trinitrobenzene sulfonic acid.

Sample	Theoretical DO	DO from TNBS Assay
**Oxi5**	5	2.8 + 0.36
**Oxi10**	10	5.1 + 1.06
**Oxi20**	20	11.2 + 1.45
**Oxi30**	30	19.4 + 1.38

## Supplementary Materials

The following are available online at https://www.mdpi.com/2073-4360/12/5/1025/s1. Figure S1: Periodate oxidation of inulin UV monitoring of the periodate oxidation of inulin reaction kinetic. Figure S2: ^1^HNMR spectra of Oxi5. Figure S3: ^1^HNMR spectra of Oxi10. Figure S4: ^1^HNMR spectra of Oxi20. Figure S5: ^1^HNMR spectra of Oxi30. Figure S6: ^1^HNMR spectra of inulin-tBC. Figure S7: ^13^CNMR spectra of inulin. Figure S8: ^13^CNMR spectra of oxidized inulin.
