# Supplementary material for: Influence of Oxidation Degree on the Physicochemical Properties of Oxidized Inulin
Source: Polymers (Basel). 2020 May 1;12(5):1025. doi: 10.3390/polym12051025 (PMC7284776; doi:10.3390/polym12051025)
Supplement: Supplementary file 1 [file polymers-12-01025-s001.pdf]

Supplementary

Influence of oxidation degree on the physicochemical properties of oxidized inulin

Franklin Afinjuomo[1], Paris Fouladian[1], Thomas G. Barclay[1], Yunmei Song[1], Nikolai Petrovsky[2,3] Sanjay Garg[1]*

[1] Pharmaceutical Innovation and Development Group, University of South Australia, Adelaide, 5000, Australia
[2] Vaxine Pty. Ltd., Adelaide, South Australia 5042, Australia
[3] Department of Endocrinology, Flinders University, Adelaide, South Australia

*Corresponding Author. Telephone: (+61) 8 8302 1567 Email: sanjay.garg@unisa.edu.au

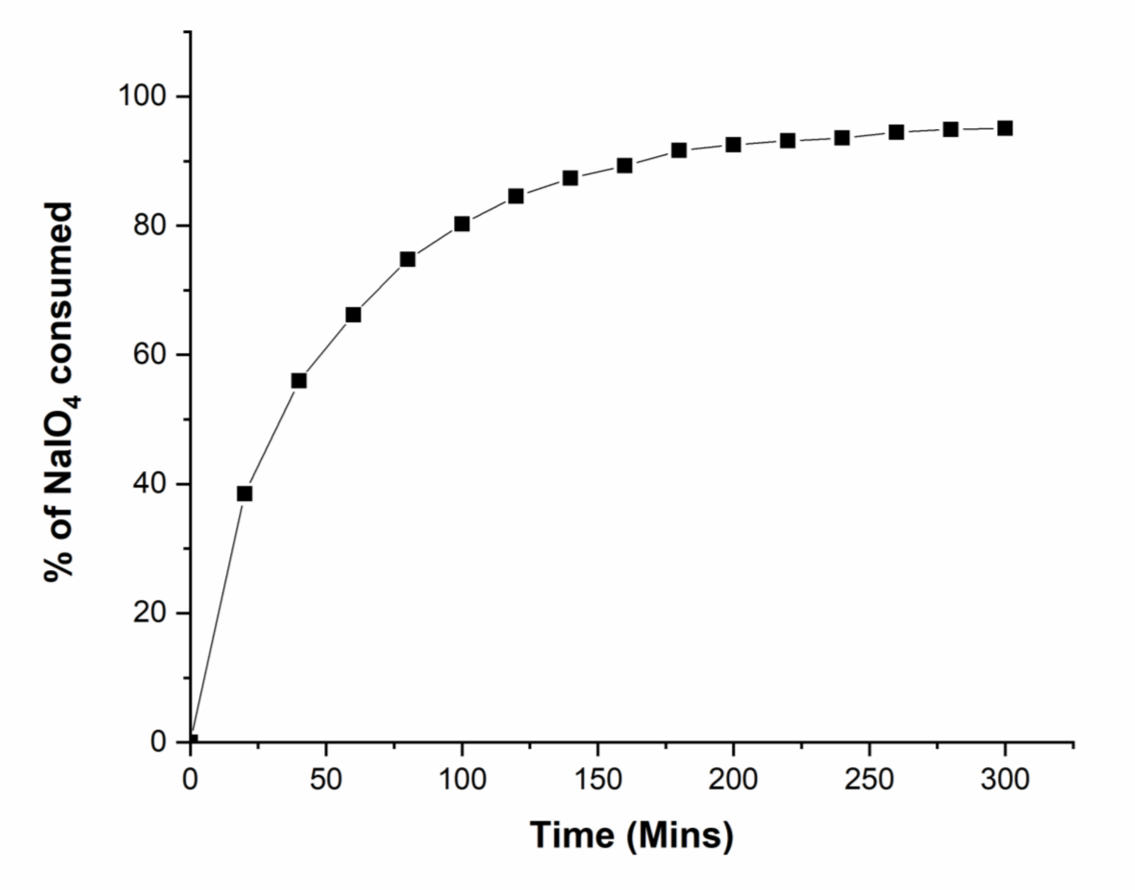

Figure S1 Periodate oxidation of inulin UV Monitoring of the Periodate oxidation of inulin

Reaction kinetic

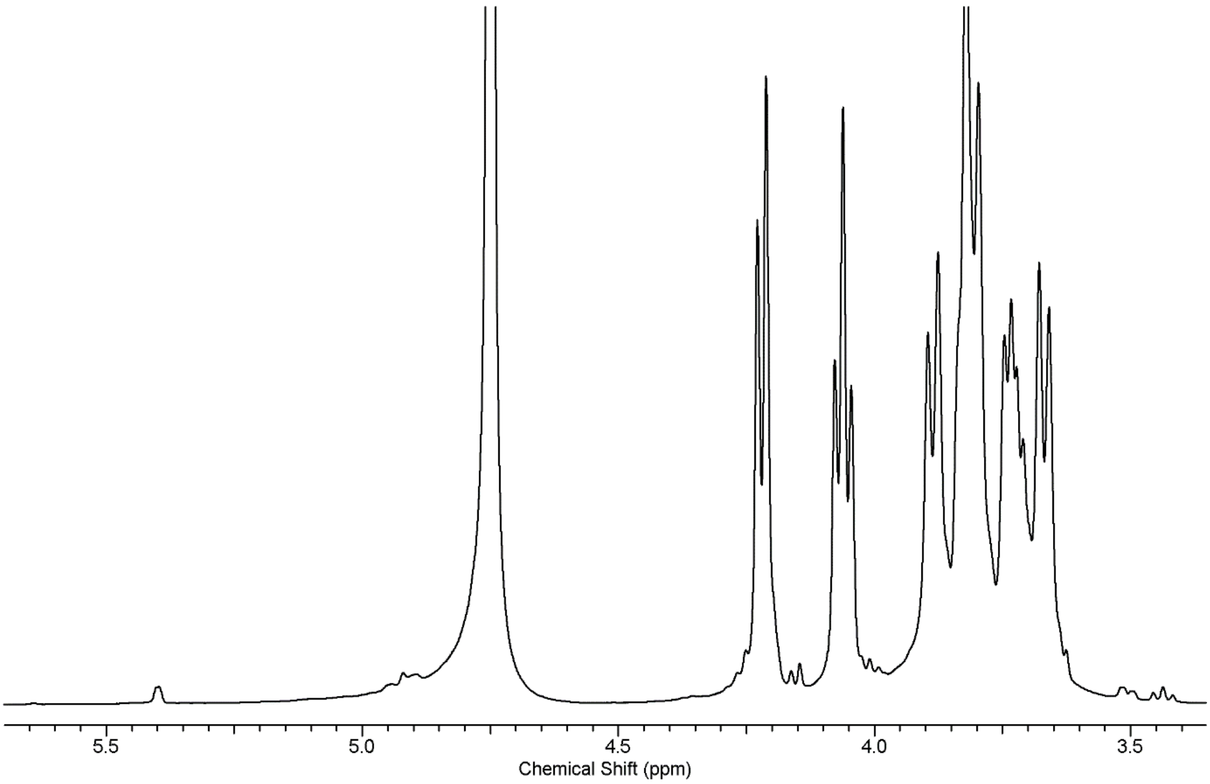

Figure S2 ^{1}HNMR spectra of Oxi 5

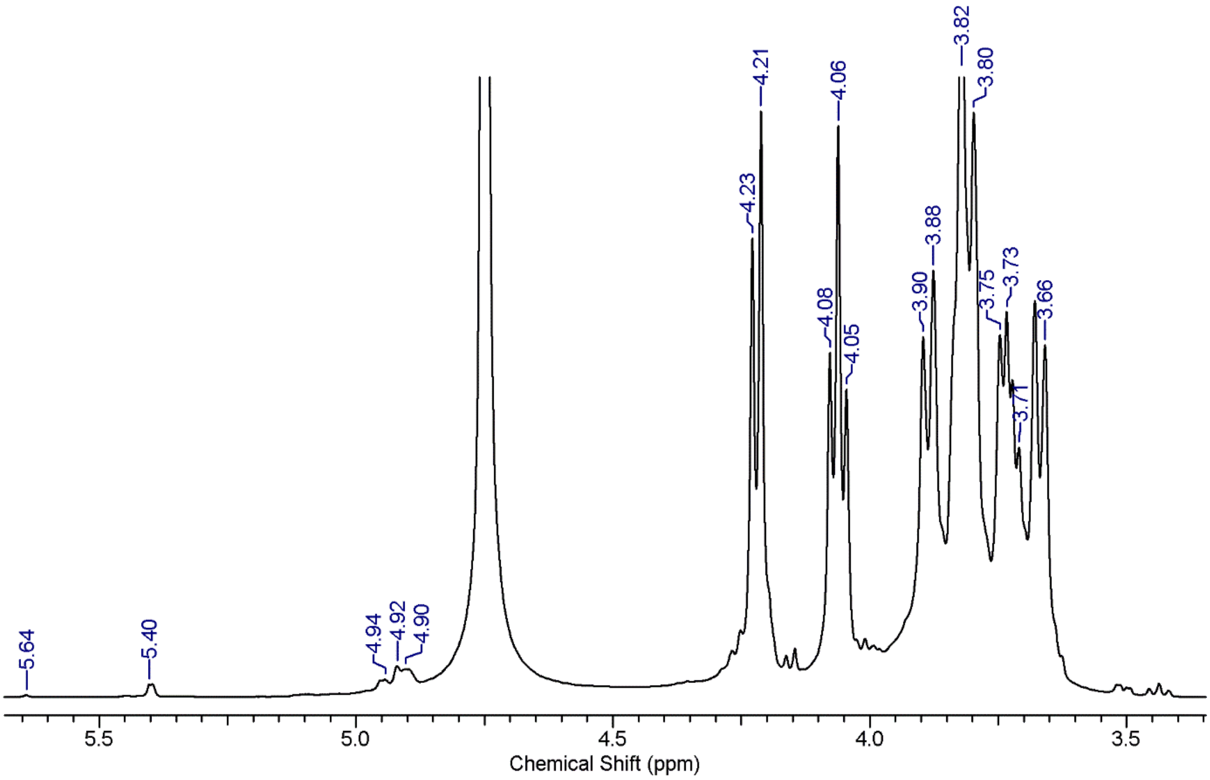

Figure S3 ^{1}HNMR spectra of Oxi 10

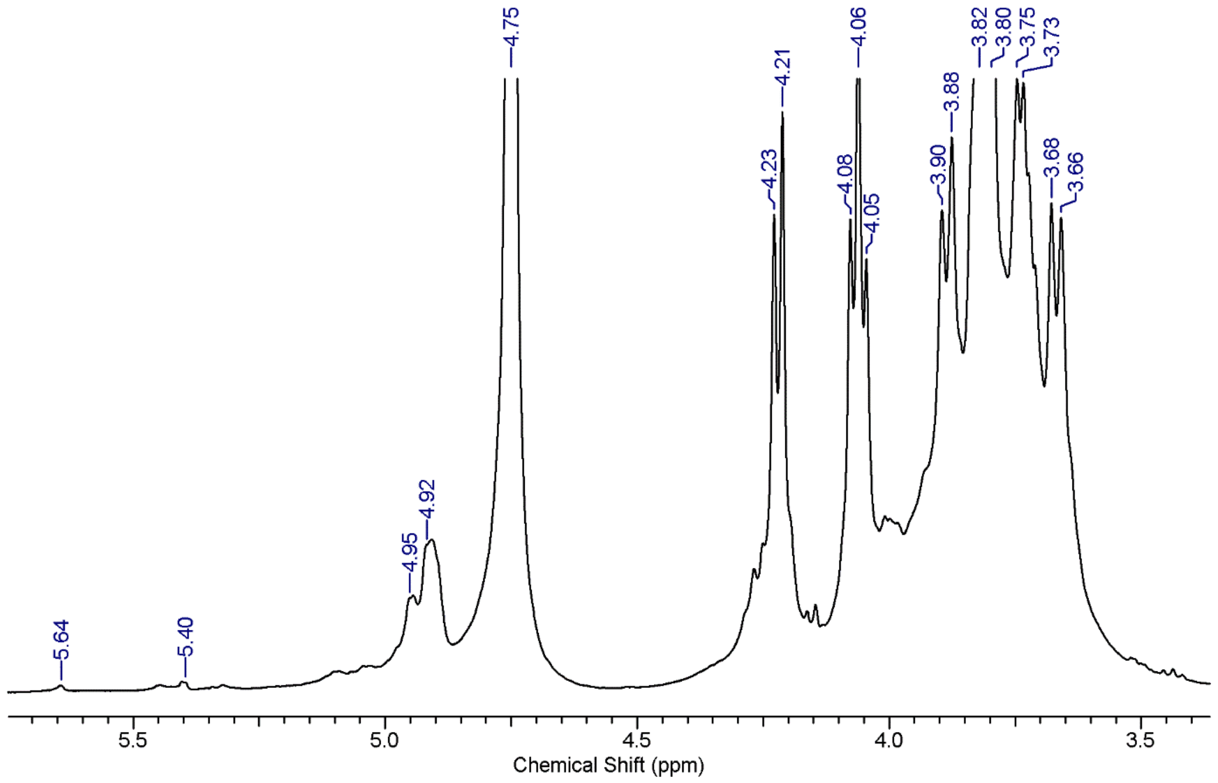

Figure S4 ^{1}HNMR spectra of Oxi 20

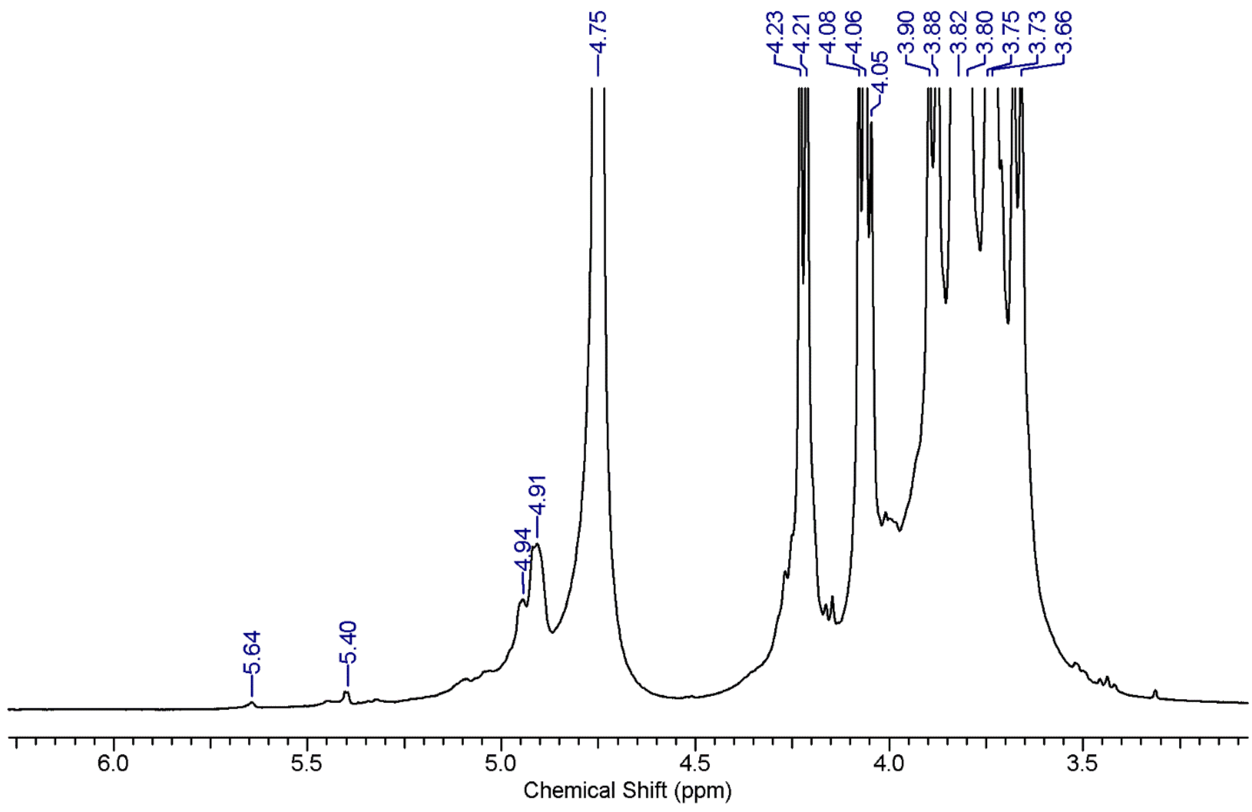

Figure S5 ^{1}HNMR spectra of Oxi 30

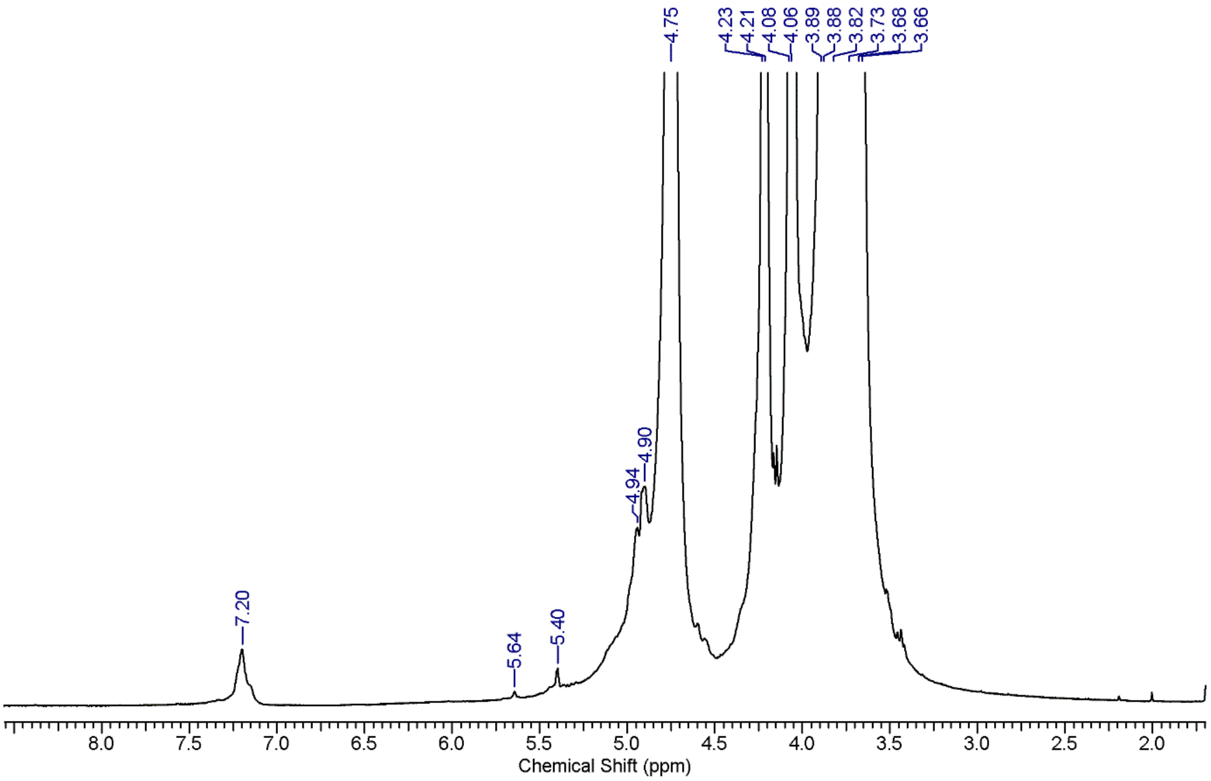

Figure S6 ¹HNMR spectra of inulin-tBC

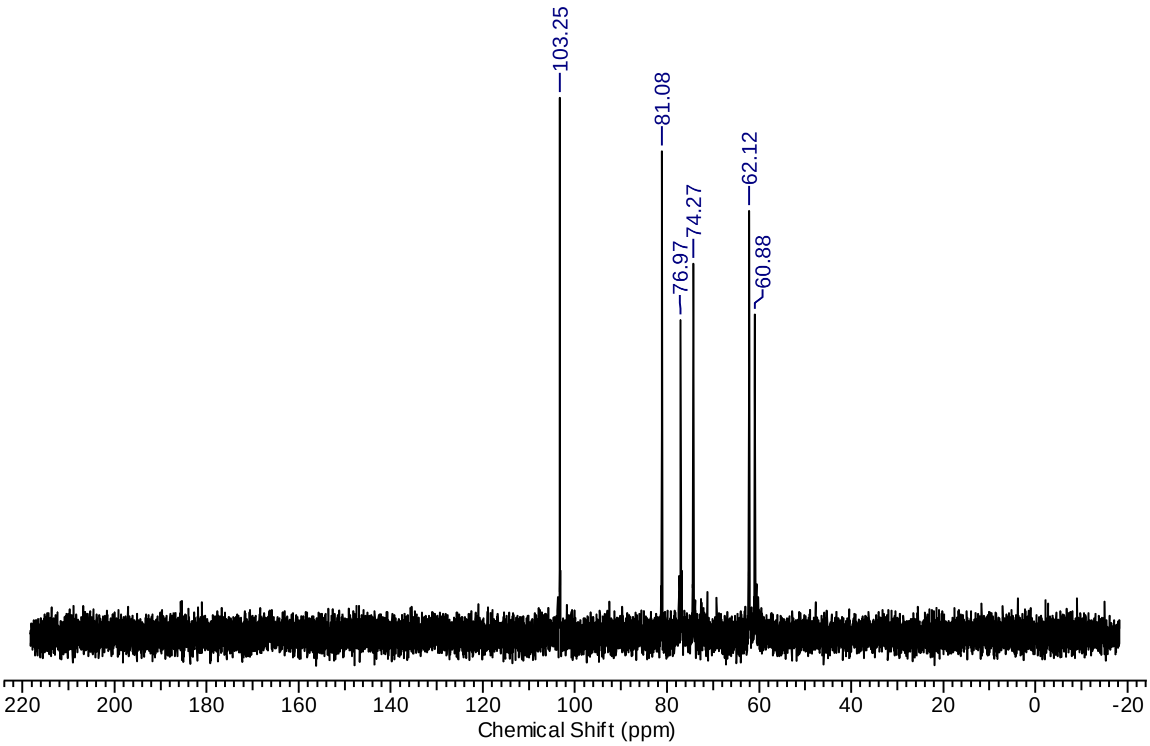

Figure S7 ¹³CNMR spectra of inulin

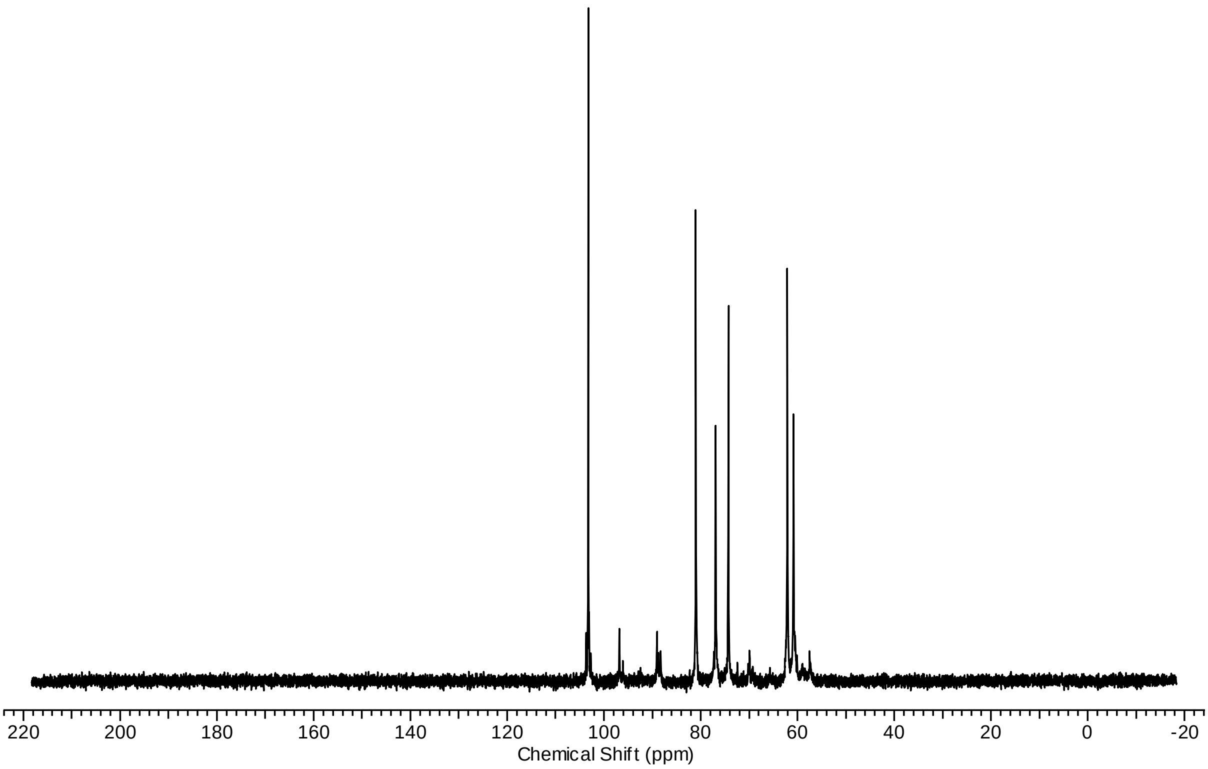

Figure S8 ^{13}CNMR spectra of oxidized inulin
